# Survival, Recruitment, and Population Growth Rate of an Important Mesopredator: The Northern Raccoon

**DOI:** 10.1371/journal.pone.0098535

**Published:** 2014-06-05

**Authors:** Elizabeth M. Troyer, Susan E. Cameron Devitt, Melvin E. Sunquist, Varun R. Goswami, Madan K. Oli

**Affiliations:** 1 School of Natural Resources and Environment, University of Florida, Gainesville, Florida, United States of America; 2 Department of Wildlife Ecology and Conservation, University of Florida, Gainesville, Florida, United States of America; CNRS, Université de Bourgogne, France

## Abstract

Populations of mesopredators (mid-sized mammalian carnivores) are expanding in size and range amid declining apex predator populations and ever-growing human presence, leading to significant ecological impacts. Despite their obvious importance, population dynamics have scarcely been studied for most mesopredator species. Information on basic population parameters and processes under a range of conditions is necessary for managing these species. Here we investigate survival, recruitment, and population growth rate of a widely distributed and abundant mesopredator, the northern raccoon (*Procyon lotor*), using Pradel’s temporal symmetry models and >6 years of monthly capture-mark-recapture data collected in a protected area. Monthly apparent survival probability was higher for females (0.949, 95% CI = 0.936–0.960) than for males (0.908, 95% CI = 0.893–0.920), while monthly recruitment rate was higher for males (0.091, 95% CI = 0.078–0.106) than for females (0.054, 95% CI = 0.042–0.067). Finally, monthly realized population growth rate was 1.000 (95% CI = 0.996–1.004), indicating that our study population has reached a stable equilibrium in this relatively undisturbed habitat. There was little evidence for substantial temporal variation in population growth rate or its components. Our study is one of the first to quantify survival, recruitment, and realized population growth rate of raccoons using long-term data and rigorous statistical models.

## Introduction

Mesopredators, or predators at middle levels of food webs, are important contributors to ecosystem function and have significant impacts on a wide range of species [1]. They can exert their influence through many means, including predation [2], competition with specialist species [3], and the spread of pathogens [4]. These effects are often heightened by the resilience and rapid growth of mesopredator populations, particularly in the presence of human settlements [5]. Basic information on mesopredator population ecology and dynamics, though crucial for developing effective conservation and management strategies, is lacking for many of these species.

One particularly abundant and widely distributed mesopredator species in the United States is the northern raccoon, *Procyon lotor*. The raccoon has proven highly successful in the past century with dramatic increases in both abundance and distribution, probably due to its skill in exploiting anthropogenic resources as well as the decimation of North American large carnivore populations [6]. In addition, the raccoon has been introduced to Japan [7], to the former Soviet Union [8], to the Queen Charlotte Islands in Canada [9], and across mainland Europe [10]. Population control efforts in these areas are hampered by insufficient knowledge of raccoon population ecology outside their native range.

As an abundant mesopredator, the raccoon has significant impacts on other species. Predation by raccoons has been shown to suppress the reproductive success of songbirds [11], waterfowl [12], and sea turtles [13], and to regulate some populations of the white-footed mouse, *Peromyscus leucopus*, [14] and the endangered Key Largo woodrat, *Neotoma floridana smalli* [15]. The raccoon also serves as a reservoir and vector for many diseases and parasites, including rabies, canine distemper, and raccoon roundworm [16]. Some of these diseases can harm less-resilient populations of other species, such as the critically endangered island fox, *Urocyon littoralis*, [17], and others can infect livestock and interfere with farming operations [18]. Because they tend to thrive in the presence of human settlements, raccoons often become nuisance animals and can even pose a threat to humans as carriers of rabies and roundworms [19], [20].

Clearly, raccoons have substantial impacts on ecology, economics, and public health, and their population dynamics are central to these impacts. However, our knowledge of their population ecology is incomplete. While raccoon survival has been estimated in many systems [21]–[24], recruitment and population growth rate have rarely or never been assessed despite being key parameters in raccoon population dynamics.

Our goal was to investigate demographic parameters and population dynamics of raccoons in a relatively undisturbed area using a detailed, long-term dataset and state-of-the-art modeling techniques. We estimated and modeled apparent survival, recruitment, and realized finite population growth rate (hereafter, population growth rate or growth rate) and tested the following hypotheses regarding these parameters: (1) Males would have lower apparent survival and higher recruitment than females, because males are more likely to disperse away from their natal population and join other populations [6]. (2) Population growth rate would covary more strongly with survival than with recruitment. Raccoons usually survive long enough to experience several breeding opportunities per lifetime, and population growth rates of species with this life history characteristic tend to be more strongly influenced by survival than reproductive rates [25]–[27].

## Materials and Methods

### Ethics Statement

All field methods followed guidelines of the American Society of Mammalogists [28] and were approved by the Animal Care and Use Committee at the University of Florida (approval number A023).

### Study Area and Species

We carried out fieldwork at the Ordway-Swisher Biological Station (OSBS), a field station located in north-central Florida (about 29.7°N and 82.0°W). OSBS is managed by the University of Florida and comprises more than 3,750 ha of protected habitat that is maintained in a fairly natural, undisturbed state. The station has a mosaic of dry and wet habitat types, including sandhills, xeric hammock, upland mixed forest, swamps, marshes, and lakes, as well as a riverine system connecting to the St. John’s River. Precipitation and temperature in the region vary seasonally–about 60 percent of annual rainfall occurs between May and September, and the temperature occasionally falls below freezing between December and March. Primary productivity, which is correlated with rainfall, is also seasonal [29]. The size of the study area remained constant throughout the period of data collection. Because the land at OSBS is protected, there was no harvesting of wildlife.

The northern raccoon is a common medium-sized carnivore across much of the United States. Some aspects of the life history and ecology of raccoons have been fairly well studied, particularly in the Midwest. In most parts of their range, the mating season lasts from January through March, peaking in February [6]. Pregnancies last around two months, resulting in a birth peak in April [6]. In the Southeast, mating generally begins later and lasts longer [6]. For example, raccoons in the mangrove swamps of southern Florida mate from March through August and give birth between May and October, with no well-defined breeding season [30]. Although no formal study has been done on breeding phenology of raccoons in north-central Florida, raccoons in this area appear to be capable of breeding year-round (M. Sunquist, pers. obs.). Litter sizes vary positively with latitude and female body size [31], commonly averaging between 3 and 4 young [32]–[34].

Females are sexually mature in the first breeding season after their birth, although yearlings usually have much lower pregnancy rates than adults [6]. If a female does not become pregnant in the regular breeding season or loses her litter soon after birth, she can experience a second estrus and produce a litter later in the year, but one litter per female per year seems to be the limit [6]. Most adult raccoons survive to breed for more than one season, but few live beyond five years [35].

### Field Methods

Raccoons were trapped once per month from September 1992 to December 1998 (76 occasions) as part of a capture-mark-recapture field study on mesopredators at OSBS. A total of 25 Tomahawk live traps were set at approximately 0.4-km intervals along a major flow-through drainage on the station. Traps were active for four consecutive nights in March and May and for two consecutive nights in all other months; we treated these multi-day trapping periods as single capture occasions in our analyses. Captured individuals were immobilized with Ketaset at a dose of 10 mg per kg of estimated body weight. They were then marked, weighed, measured, sexed, and released at the capture location. Raccoons were initially radio-tagged with a small tag in each ear. Starting in the second year of the study, due to a few instances of tag loss, raccoons were instead marked with a microchip that was inserted just under the skin on the back of the neck.

### Capture-Mark-Recapture Analyses

We used two parameterizations of Pradel’s [36] temporal symmetry model to estimate and model survival, recruitment, and population growth rate. One (φ and λ parameterization) estimates apparent survival probability (φ), recapture probability (*p*), and realized population growth rate (λ); the other (φ and *f* parameterization) estimates recruitment (*f*) rate instead of population growth rate. We carried out all analyses using program MARK [37] version 6.2 implemented through the RMark package for program R [38] version 2.15.2. For model selection, we used an information-theoretic approach with Akaike’s information criterion corrected for small sample size (AIC_c_) to determine model parsimony and make statistical inferences [39], [40]. We treated all raccoons within the study area as part of one open population.

Pradel’s model is sensitive to trap response, or differences in capture probabilities of marked vs. unmarked individuals [41]. Several raccoons in this study appeared to be “trap happy” and repeatedly reentered traps to retrieve bait (M. Sunquist, pers. obs.). To reduce bias caused by trap response, we removed all individuals that were caught on 5 or more consecutive occasions from the data.

We ran two model sets to determine the best model structure for each parameter: one using the φ and λ parameterization and one using the φ and *f* parameterization. In each set, we tested for the effects of sex, month, season, and additive and interactive effects between sex and the other variables on each parameter (recapture probability, apparent survival, recruitment rate, and population growth rate). We parameterized our models in RMark using fully time-dependent parameter index matrices (PIMs); models were constrained using the design matrix. Because data were collected in monthly intervals and all models were parameterized using time-dependent PIMs, all estimates of model parameters were monthly, regardless of the model structure. We considered four biologically relevant seasons based on preliminary analyses and climatic patterns of the region: March–May (spring), June–August (summer), September–November (fall), and December–February (winter).

We tested goodness of fit using RELEASE TEST 2+3. We found no evidence for lack of fit or overdispersion (χ^2^_346_ = 252.58, *P* = 1.00, *ĉ* = 0.73).

## Results

There were 1,095 captures of 348 raccoons over the course of the field study. Of these individuals, 7 were captured on 5 or more consecutive occasions and were therefore excluded from analyses. The final dataset included 983 captures of 341 raccoons, with 343 captures of 112 females and 640 captures of 229 males. The average number of captures per individual was close to 3 for both sexes, but overall there were twice as many males in the study as females.

### Recapture Probability

**Figure 1:**
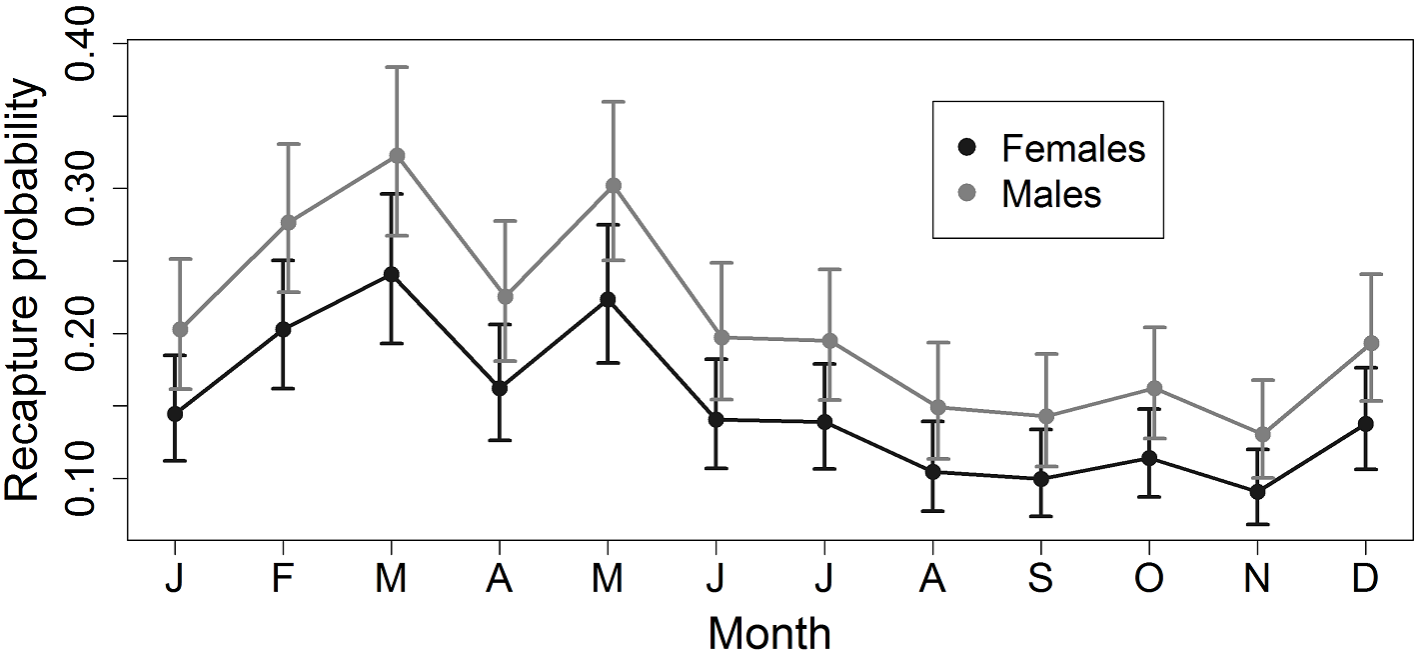
Monthly recapture probability of northern raccoons (*Procyon lotor*) at the Ordway-Swisher Biological Station, Florida. Estimates and 95% confidence intervals are from model {φ(sex)*p*(month+sex)λ(season)}.

The best model for recapture probability, with a cumulative weight of 0.809 in the recruitment model set and 0.897 in the lambda model set, included an additive effect of month and sex (Table 1). Monthly recapture probabilities based on this model were consistently higher for males than females (Figure 1); estimates from model {φ(sex)*p*(month+sex)λ(season)} were highest in March (0.323, 95% CI = 0.267–0.384 for males; 0.241, 95% CI = 0.193–0.296 for females) and lowest in November (0.130, 95% CI = 0.100–0.168 for males; 0.091, 95% CI = 0.068–0.120 for females). The peaks in recapture probability during March and May probably result from the longer capture period in those months (4 nights of trapping compared with 2 nights for all other months; see Methods).

**Table 1:** Model comparison table for Pradel’s temporal symmetry analysis of data collected from a population of northern raccoons (*Procyon lotor*) at the Ordway-Swisher Biological Station, Florida, from September 1992 to December 1998.

Model	K	AIC_c_	ΔAIC_c_	Weight	Deviance
**(A) Recruitment parameterization**					
φ(sex)*p*(month+sex)*f*(season+sex)	20	6885.994	0.000	0.486	3540.180
φ(sex)*p*(month+sex)*f*(sex)	17	6888.242	2.248	0.158	3548.667
φ(sex)*p*(season+sex)*f*(sex)	9	6889.819	3.826	0.072	3566.694
φ(season+sex)*p*(month+sex)*f*(season+sex)	23	6889.988	3.994	0.066	3537.896
φ(sex)*p*(month+sex)*f*(season×sex)	23	6890.363	4.369	0.055	3538.271
φ(sex)*p*(season+sex)*f*(season+sex)	12	6891.611	5.618	0.029	3562.349
φ(season+sex)*p*(month+sex)*f*(sex)	20	6892.186	6.192	0.022	3546.372
φ(sex)*p*(season×sex)*f*(sex)	12	6892.196	6.202	0.022	3562.933
φ(sex)*p*(season×sex)*f*(season+sex)	15	6893.417	7.424	0.012	3557.981
φ(sex)*p*(month×sex)*f*(season+sex)	31	6894.266	8.273	0.008	3525.239
**(B) Lambda parameterization**					
φ(sex)*p*(month+sex)λ(season)	19	6882.462	0.000	0.380	3538.732
φ(sex)*p*(month+sex)λ(season+sex)	20	6883.774	1.312	0.197	3537.960
φ(season+sex)*p*(month+sex)λ(season)	22	6884.476	2.014	0.139	3534.481
φ(season+sex)*p*(month+sex)λ(season+sex)	23	6885.834	3.372	0.070	3533.742
φ(sex)*p*(month+sex)λ(.)	16	6887.068	4.606	0.038	3549.564
φ(sex)*p*(month+sex)λ(sex)	17	6888.242	5.780	0.021	3548.667
φ(sex)*p*(season+sex)λ(.)	8	6888.675	6.213	0.017	3567.587
φ(sex)*p*(month+sex)λ(season×sex)	23	6888.777	6.315	0.016	3536.685
φ(sex)*p*(season+sex)λ(season)	11	6888.990	6.528	0.015	3561.778
φ(sex)*p*(season+sex)λ(sex)	9	6889.819	7.357	0.010	3566.694

We ran model sets investigating the effects of sex, time, month, season, and additive and interactive effects of sex with the other variables on four parameters: recapture probability, *p*; apparent survival probability, φ; recruitment rate, *f*; and realized population growth rate, λ. In section (A), we used the recruitment parameterization; in section (B), we used the lambda parameterization. Only the top ten models are included for each set. K is the number of parameters, AIC_c_ is Akaike’s Information Criterion corrected for small sample size, ΔAIC_c_ is the difference between each model’s AIC_c_ and the AIC_c_ of the top-ranked model, weight is the Akaike weight or model probability, and deviance is the model deviance.

### Apparent Survival Probability

All well-supported models (ΔAIC_c_ <14 under both parameterizations, Table 1) included an effect of sex on survival probability, providing strong evidence that monthly survival differed between sexes. The top model for survival, with a cumulative weight of 0.857 in the recruitment model set and 0.737 in the lambda model set, included no temporal variation in survival. Estimates of monthly survival from model {φ(sex)*p*(month+sex)λ(season)} were 0.949 for females (95% CI = 0.936–0.960) and 0.908 for males (95% CI = 0.893–0.920). Annual survival based on these monthly values was 0.534 for females (95% CI = 0.452–0.610) and 0.313 for males (95% CI = 0.258–0.370).

**Figure 2:**
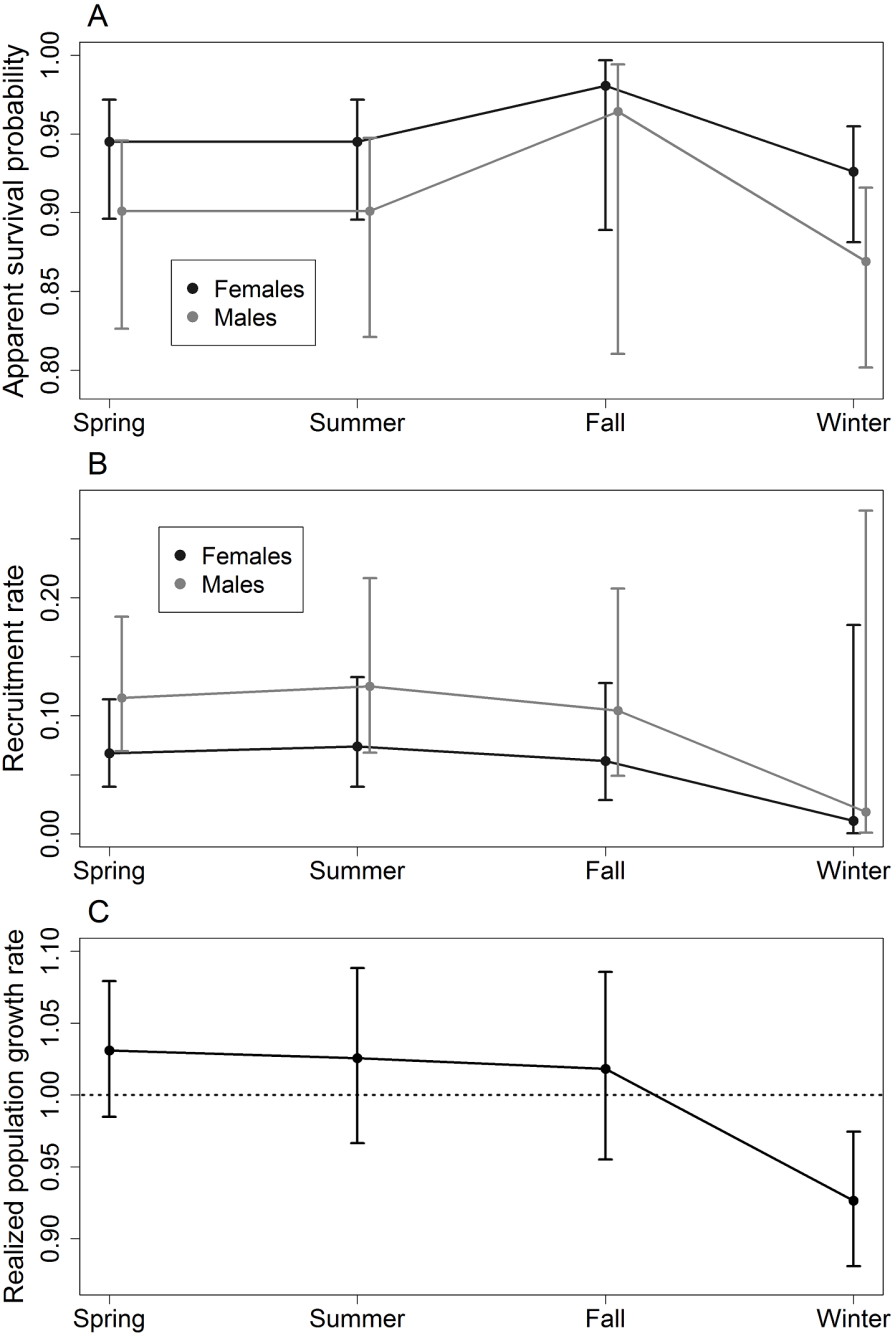
(A) Monthly apparent survival probability, (B) monthly recruitment rate, and (C) monthly realized population growth rate of northern raccoons (*Procyon lotor*) at the Ordway-Swisher Biological Station, Florida. Estimates and 95% confidence intervals are from the top-ranked model that included season for each parameter: {φ(season+sex)*p*(month+sex)λ(season)} for survival, {φ(sex)*p*(month+sex)*f*(season+sex)} for recruitment, and {φ(sex)*p*(month+sex)λ(season)} for population growth rate.

The second-best model for survival, with a cumulative weight of 0.110 in the recruitment model set and 0.247 in the lambda model set, included an additive effect of season and sex. Estimates of monthly survival from model {φ(season+sex)*p*(month+sex)λ(season)} are shown in Figure 2A. However, the 95% confidence intervals of these estimates overlapped for all seasons, and the 95% confidence intervals for the β parameters of all seasons included zero, indicating little evidence for seasonal variation in survival.

### Recruitment Rate

Monthly recruitment rate differed between sexes (ΔAIC_c_<12, Table 1A). The best-supported model, which had a cumulative weight of 0.629, included an additive effect of sex and season. Estimates of monthly recruitment from model {φ(sex)*p*(month+sex)*f*(season+sex)} were consistently higher for males than for females (Figure 2B), peaking in summer (0.125 for males, 95% CI = 0.069–0.217; 0.074 for females, 95% CI = 0.040–0.133).

Although {φ(sex)*p*(month+sex)*f*(season+sex)} was the top model, the 95% confidence intervals for the β parameters of all seasons included zero, and the 95% confidence intervals for the recruitment estimates from this model overlapped between all seasons. These confidence intervals suggested little or no evidence for discernible seasonal variation in recruitment rate. The second-best model for recruitment (cumulative weight = 0.292) included an effect of sex with no temporal variation, further suggesting that recruitment rate did not vary substantially over time. Monthly recruitment estimates from model {φ(sex)*p*(month+sex)*f*(sex)} were 0.091 for males (95% CI = 0.078–0.106) and 0.054 for females (95% CI = 0.042–0.067).

### Realized Population Growth Rate

The best-supported model for population growth rate included an effect of season and had a cumulative weight of 0.554 (Table 1B). Estimates of monthly growth rate from model {φ(sex)*p*(month+sex)λ(season)} are shown in Figure 2C; the highest estimate was 1.031 (95% CI = 0.985–1.079), which occurred in spring. Model {φ(sex)*p*(month+sex)λ(season+sex)} had a ΔAIC_c_<2 and a cumulative weight of 0.286 (Table 1B), suggesting a sex difference in population growth rate in addition to seasonal variation. However, in both these top models, the 95% confidence intervals of all β parameters for season and sex included zero. These confidence intervals indicate little evidence that population growth rate varied seasonally or between sexes.

Consistent with this lack of compelling evidence for any variation in population growth rate, the third-best supported model for growth rate was the constant parameter model. This model, {φ(sex)*p*(month+sex)λ(.)}, estimated a monthly population growth rate of 1.000 (95% CI = 0.996–1.004). The annual population growth rate derived from this estimate was 0.997 (95% CI = 0.948–1.050). A realized population growth rate of 1 means that the population size is neither increasing nor decreasing, indicating that our study population may have reached a stable equilibrium.

Based on the confidence intervals of effect sizes and real estimates, as described in the preceding sections, there was little evidence that population growth rate or its components (survival and recruitment) varied substantially over time. We show seasonal estimates of these parameters in Figure 2 to give an idea of what the temporal dynamics might be, but differences in parameter estimates between seasons are slight.

## Discussion

Mesopredators constitute one of the most ecologically influential groups of vertebrates in the modern world, but research on their population ecology is remarkably scarce and limited in scope and often uses coarse-scale data [24], [42]. Our goal in this study was to report rigorous estimates of survival, recruitment, and population growth rate for a ubiquitous mesopredator, the northern raccoon, based on a long-term dataset. We found that raccoon population parameters in our relatively undisturbed system varied little over time, indicating a fairly stable population size.

Consistent with our hypothesis, male raccoons had lower apparent survival and higher recruitment, probably due to the permanent immigration and emigration of males to and from the study area associated with natal and breeding dispersal. The same pattern has been found for Virginia opossums (*Didelphis virginiana*) in this system, an ecologically similar mesopredator species that also exhibits male-biased natal and breeding dispersal [43]. In other mesopredator populations, sex differences in survival are often not present. Clearly, the conditions in each system and life history characteristics of each species (e.g., cost of reproduction, differential parental investment) influence whether one sex will have higher survival than the other, but most studies do not investigate what those factors are [21], [23], [42].

Precipitation and temperature determine primary productivity and thus also influence higher levels of food webs. Because rainfall and temperatures in our study site vary over time, we expected evidence of temporal variation in some or all of the population parameters we studied. Many other studies have found links between population dynamics and climatic variables [44]–[46]. However, we found little evidence that either population growth rate or its components varied substantially over time. Raccoons are highly adaptable in terms of diet and habitat tolerance, and the climate in Florida is relatively mild with a low range of variation. These factors likely minimized any temporal variation in raccoon population dynamics that might have resulted from climatic variation.

In contrast, a sympatric population of Virginia opossums showed monthly variation in population growth rate and recruitment, strong covariation between these parameters, and a positive association of these parameters with precipitation variability (coefficient of variation of rainfall) [43]. While opossums and raccoons are ecologically similar as mesopredators, they follow different life history strategies–opossums live a faster life, with rapid reproduction and early senescence; raccoons live a slower life, with smaller litters spaced over longer lifespans [27]. In addition, opossums have clearly-defined breeding seasons [29], while Florida raccoons can apparently breed year-round (M. Sunquist, pers. obs.). These different strategies appear to have resulted in distinct population dynamic patterns. In general, population size is less temporally variable in species with slower life histories than in those with faster life histories. The relative influence of vital rates on population growth rate also depends on life history strategy, in that population growth rate tends to be more sensitive to survival in slow species and to recruitment in fast species [25], [47]. This example further illustrates the importance of detailed studies on the population ecology of individual mesopredator species. Even though these species often have similar diets, habitat preferences, and levels of human tolerance, dissimilarities in their reproductive schedules can cause their populations to function differently.

Although some aspects of raccoon ecology have been studied intensively [33], [48]–[51], there has been little focus on raccoon population dynamics and the factors and processes underlying population growth rate. This study is the first to provide rigorous estimates of population growth rate and its underlying vital rates for raccoons in a natural habitat, and the results suggest that this undisturbed population was at a stable equilibrium. For many other mesopredator species, basic population ecology is poorly understood, which can hamper efforts to determine drivers of mesopredator population dynamics and factors that allow mesopredators to thrive in a variety of habitats. As human impacts continue to grow in magnitude around the world [52]–[54], human-tolerant mesopredators will continue to increase in distribution and impact as well, with potentially devastating consequences for biodiversity [1], [55]. Comparative studies of mesopredator population ecology in relatively undisturbed versus heavily disturbed habitats, including some quantification of mesopredator impacts on communities in each habitat type, would allow us to better understand how mesopredator populations respond to human disturbance and how these responses might affect community structure in general.
